# Opium alkaloids, biosynthesis, pharmacology and association with cancer occurrence

**DOI:** 10.1098/rsob.220355

**Published:** 2023-05-03

**Authors:** Agrataben Vadhel, Sabreen Bashir, Ashiq Hussain Mir, Madhuri Girdhar, Deepak Kumar, Anil Kumar, Aradhana Mohan, Tabarak Malik, Anand Mohan

**Affiliations:** ^1^ School of Bioengineering and Biosciences, Lovely Professional University, Phagwara 144411, India; ^2^ School of chemical engineering and Physical sciences, Lovely Professional University, Phagwara 144411, India; ^3^ Gene Regulation Laboratory, National Institute of Immunology, New Delhi 110067, India; ^4^ Department of Biomedical Engineering, University of Michigan, Ann Arbor, MI 48109, USA; ^5^ Department of Biomedical Sciences, Institute of Health, Jimma University 00000, Ethiopia

**Keywords:** opioid, biosynthetic pathway, phytochemistry, pharmacology, tumour

## Abstract

*Papaver somniferum* L. (Family: Papaveraceae) is a species well known for its diverse alkaloids (100 different benzylisoquinoline alkaloids (BIAs)). L-tyrosine serves as a precursor of several specific metabolites like BIAs. It has been used as an antitussive and potent analgesic to alleviate mild to extreme pain since ancient times. The extraction of pharmaceutically important alkaloids like morphine and codeine from poppy plant reflects the need for the most suitable and standard methods. Several analytical and extraction techniques have been reported in open literature for morphine, codeine and other important alkaloids which play a vital function in drug development and drug discovery. Many studies suggest that opioids are also responsible for adverse effects or secondary complications like dependence and withdrawal. In recent years, opium consumption and addiction are the most important risk factors. Many evidence-based reviews suggest that opium consumption is directly linked or acts as a risk factor for different cancers. In this review, we highlight significant efforts related to research which have been done over the past 5 decades and the complete information on *Papaver somniferum* including its phytochemistry, pharmacological actions, biosynthetic pathways and analytical techniques of opium alkaloid extraction and the link between opium consumption and cancer-related updates.

## Graphical abstract

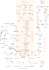

## 1. Introduction

Alkaloids are defined as nitrogen-containing heterocyclic compounds, found in the plant kingdom. Various types of alkaloids in plant families have different positions for the nitrogen atom. Some of the plant families are notably high in alkaloid content such as Papaveraceae, Solanaceae, Amaryllidaceae and other well-known families [1,2]. Opium poppy which is botanically known as *Papaver somniferum* belongs to the family Papaveraceae. Opium is air-dried milky latex or resin obtained from the seed pod of the poppy plant [3]. Its latex is a rich source of benzylisoquinoline alkaloids (BIAs) which are accumulated in laticifer (a specialized internal secretory cell). It is also popular due to its attractive flower and seeds which are commonly used in confectioneries [4,5]. Opium alkaloids can be classified into three main classes: natural, semi-synthetic and synthetic alkaloids. Opiates are the active ingredients derived naturally from opium. Besides morphine, there are several well-known synthetics or semi-synthetic opium derivatives including substances similar to these opiates such as heroin (diacetylmorphine), methadone, hydrocodone (in Lortab, Norco, Vicodin), oxycodone (OxyContin), fentanyl and others. These compounds are collectively known as opioids [6].

Since ancient times, opium has been harvested in various countries for recreational or medicinal purposes [7,8]. India is the only legal opium exporter in the world with its fourth position for production, which is managed with strict control by the government [9]. It contains more than 30 different types of alkaloids, so it is necessary to extract and identify each bioactive compound. From ancient times researchers have been using many techniques for the separation of alkaloids so that they can be used for medicinal purposes. It reveals that opium alkaloid can be used in the drug industry to cure analgesic, microbial, inflammatory and neurological ailments [10]. On other hand, various studies suggest that opioids affect the brain's reward function by altering the molecular and neural pathways of brain. The use of opioid leads to neuroadaptations and can trigger changes to the brain's emotional and pain systems, resulting in hypersensitivity and a higher risk of compulsive drug-seeking behaviour. Long lasting use of opioid use causes changes to the structure and function of the brain, mainly in regions involved in reward, motivation and decision-making. These changes can affect neural circuitry and neurotransmitter systems, resulting in cognitive impairment and an increased risk of mental health disorders such as anxiety and depression. In addition, it can have significant psychological effects such as changes in behaviour and mood. It can induce feelings of euphoria and cause social and occupational damages such as withdrawal from family and friends, loss of employment and financial instability [11,12]. Apart from the psychological and neurological impact of opioids, many studies in recent years are drawing the global attention towards possible connection between opium consumption and tumour formation.

The correlation between the risk of tumour formation and opium consumption has been reported by many studies [13,14]. The positive and negative effect of opium is still a controversial matter for the last few years [15,16]. Multiple articles of cellular and biological *in vivo* [17] and *in vitro* [18] reports prove that opioids may directly or indirectly promote cancer. Thus, the role of morphine in neoplasia is still contradictory. On the other hand, a lot of research done on cancer cell lines and animal models depict that opium alkaloid morphine can suppress the growth of various types of cancers. Unfortunately, the mechanism of tumour cell growth regulation and the effect of morphine on it, is not yet correctly established. Tumour growth is a multi-step process that results from changes at the gene level causing normal cells to transform into malignant cells. The impact of opium alkaloids on tumour formation has been discussed in many studies. This review intends to focus on phytochemistry and a comprehensive view of various analytical techniques, pharmacology and their effect on human health.

## 2. Extraction and analytical techniques of different alkaloids present in poppy

In recent years, due to advancement in crude drugs and chemical composition knowledge, various methods like biochemical, biological and spectroscopy have been used for evaluating active compounds. Different methods are used for the evaluation and characterization of morphine and related alkaloids from poppy straw. Analytical techniques include several procedures such as crude drug or dried powder collection, extraction and analysis of the sample, metabolite compound separation handling and quantification for statistical purposes [19]. The extraction of samples acts as an essential step in the analysis, especially in chromatographic analysis procedures. The extraction techniques have been classified into two ways: conventional or contemporary and non-conventional or green extraction methods. The effect of bioactive compound extraction mostly depends on the origin or nature of plant material, moisture content, particle size and the degree of processing. Moreover, the quantity of extractive yield composition depends on the different parameters such as the nature of the compound, the concentration of solvent and its polarity, temperature and extraction time; in the case of opium alkaloid identification, it has been reported that with correct extraction and analytical techniques, opium alkaloid trace identification was possible on archaeological ceramic vessels [20].

Nowadays, the alkaloids are extracted from dried plant powder using an ultrasonic-assisted extraction method which is suggested as an efficient extraction process as it enhances the mass transfer of bioactive compounds by disrupting the cell wall [21]. There are various chromatographic techniques by which opium alkaloids can be identified. For the detection and separation of a specific compound, high-performance liquid chromatography (HPLC) is more often applicable. It is a widely used technique for industrial and scientific purposes like forensic, chemical and pharmaceutical analysis. Besides HPLC, the other methods which are used for the analysis of the alkaloids present in opium include HPTLC and GC (table 1).

**Table 1.** Extraction and analysis of alkaloids from *Papaver somniferum.*

analyte	extraction technique	analytical technique	instrument condition	detection mode	limit of quantization (LOQ)/ limit of detection (LOD)	references
morphine, codeine, oripavine, thebaine, narceine	10 gm sample in 100 ml acetonitrile: water: formic acid	LC-MS/MS	C-18 column, ammonium carbonate—eluent A and acetonitrile—eluent B.	(ESI+) and (MRM)	n.a.	[5]
opium alkaloids	ultrasonic-assisted extraction (UAE)	capillary electrophoresis	fused-silica capillary 60 cm × 50 µm, temperature—251C, voltage—20 kV	UV detector, 214 nm	LOD − 0.2 mg mL^−1^ LOQ − 2 mg mL^−1^	[21]
morphine, codeine, oripavine, thebaine, noscapine	ultrasonic-assisted extraction temperature—40°C time—20 min.	RP-HPLC	column—C-8 sodium heptane sulfonate—mobile phase final quantification—C-18 column	DAD, 280 nm	LOD (3.3*σ*/S) (MO- 1.8, OR-0.3, CD-0.6, PA- 0.3, TH-0.2, NS-0.5) μg mL^−1^.	[22]
					LOQ (10 *σ*/S) (MO- 5.4, OR-0.9, CD-1.8, PA-0.8, TH-0.6 NS1.6) μg mL^−1^	
morphine, codeine, oripavine, thebaine, noscapine	1 gm powder capsule extraction in methanol	HPLC	column—C-18 (5 µm, 250 mm × 4.6 mm),	PDA detector, 240 nm	n.a.	[23]
			acetonitrile-phosphate buffer-glacial acetic acid—mobile phase, column temperature, −26°C,			
			1.0 mL/ min—flow rate pH-3.8			
morphine, codeine, oripavine, thebaine	ultrasonic agitation- 1 gm dried capsule powder 10 ml methanol. temp—40−45°C.	HPLC	column—C-18	PDA detector, 240 nm	n.a.	[24]
			(5 µm, 4.6 × 250 mm),			
			sodium phosphate buffer—mobile phase-A			
			acetonitrile—mobile phase-B			
			ml per min—flow rate pH-3.			
morphine, codeine, oripavine, thebaine, noscapine	solid-phase extraction (SPE) 50 mg plant material with 5 ml of 5% acetic acid	HPLC	column—F5 (5 µm, 150 mm × 4.6 mm) 5% of acetonitrile—mobile phase A—acetonitrile: glacial acetic acid: triethylamine— mobile phase-B	UV/VIS and fluorescence, 284 nm	LOD (MO-1.28 μg mL^−1^ (0.013%). LOQ (MO-4. μg Ml^−1^ (0.043%).	[25]
morphine	ultrasonic-assisted extraction (UAE)	HPLC	column—C-18, 0.1% TFA in water		LOD 1.8	[26]
			solvent-A-TEA		LOQ 5.4	
			solvent-B-methanol			
noscapine	crude extract poppy straw	HPLC	column—C-18 5 µm, 120 mm × 4 mm,	UV-vis detection	n.a.	[27]
			methanol:acetonitrile:water—mobile phase			
			temperature—40°C			
			1.0 mL/min—flow rate			
morphine, codeine, oripavine, thebaine, noscapine	solid-phase extraction (SPE)-A poppy straw extraction with 5% acetic acid under sonication	HPLC-MS/MS	HyPURITY AQUASTAR column		LOD-(MO-2.6,	[28]
			formic acid in methanol—mobile phase-A,		CD-1.6, NR-0.4, PA- 0.4	
			formic acid in deionized water—mobile phase-B		TH- 17.5 (μg g^−1^)	
					LOC- (MO- 7.8, CD-	
					4.8, NR-1.3, PA- 1.1.	
					TH 52.2 (μg g^−1^).	
morphine, codeine, oripavine, thebaine, noscapine	1 gm capsule husk in methanol	HPTLC	silica gel plates 60 F254, mobile phase-toluene- acetonemethanol-ammonia (40:40:6:2) v/v/v	Dragendorff reagent, 540 nm.	n.a.	[29]
thebaine	75 mg powder in 10 ml DMSO	HPLC	column—C-18, methanol:glacial acetic acid: Millipore water (40:1:59)v/v/v- Mobile phase	dual-absorbance detector, millennium32 software.	n.a.	[30]
morphine	n.a.	FT-IR spectra	Raman Scope III instrument diode-pumped Nd: YAG (neodymium-doped yttrium aluminium garnet)	laser emitting at 1064 nm (laser power of 100 mW).	n.a.	[31]
thebaine	ultrasonic bath	RP-HPLC	column—C-18 monolithic (100 mm × 4.6 mm × 5 µm),	UV-VIS detector, 285 nm	n.a.	[32]
			trifluoroacetic acid and formic acid—mobile phase-A			
			trifluoroacetic acid and formic acid in acetonitrile—mobile phase-B		
		GC	column-VF-5MS	mass selective detector	n.a.
			(0.25 µm, 30 m × 0.25 mm)		
			helium—carrier gas		
			temperature—260°C			
opium alkaloids	solid-phase extraction	RP-HPLC	column—C-18	UV detection, 283 nm.	n.a.	[33]
			(3.0 mm × 150 mm; 3.5 mm),			
			water (H_2_O/triethylamine)^-^ mobile phase-A methanol (CH_3_OH/triethylamine)			
morphine, codeine, oripavine, noscapine	n.a.	RP-HPLC (MLC)	column—C-18 (150 mm × 4.6 mm, 5 µm),	spectrophotometric detector	LOD-3.3 s/m LOQ-10 s/m	[34]
morphine		GC-MS	silica capillary column, carrier gas—helium, column (5 µm, 30 m × 0.25 mm)	detector voltage −1700	n.a.	[35]

## 3. Biosynthetic pathway

**Figure 1.**
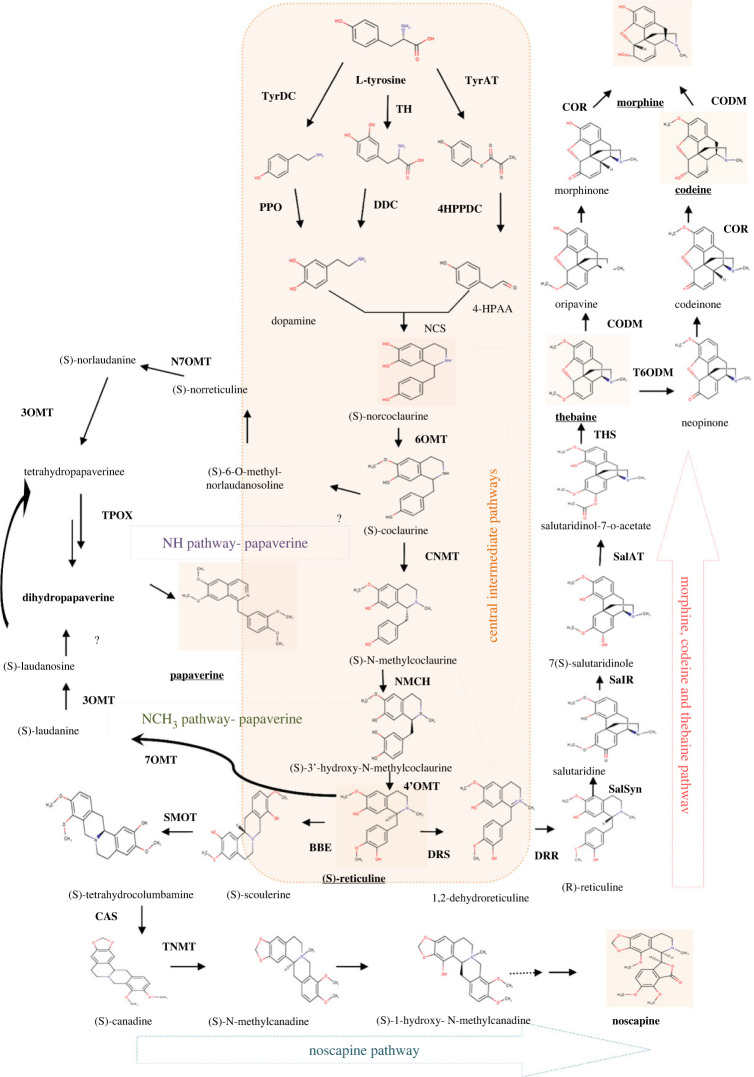
The schematic biosynthetic pathway of major alkaloids in *Papaver somniferum* begins with central intermediate (S)-reticuline leading to papaverine with two hypothetical pathways—NH pathway (purple) and NH_3_ pathway and Noscapine (green)—and a common pathway of morphine, codeine and thebaine. The pathway has been deduced and compiled from Han *et al*. [36], Pathak *et al*. [37], Rezaei *et al*. [38] and Singh *et al*. [39]. Abbreviation: TyrDC—tyrosine decarboxylase, TH—tyrosine hydroxylase, TyrAT—tyrosine transaminase, PPO—polyphenol oxidase, DDC—DOPA decarboxylase, 4-HPPDC—4-HPP decarboxylase, 4-HPAA—4-hydroxyphenylacetaldehyde, NCS—norcoclaurine synthase, 6OMT—6-O-methyltransferase, CNMT—coclaurine N-methyltransferase, NMCH—(S)-N-methyl coclaurine 3-hydroxylase, 4 OMT—4-O-methyltransferase, DRS—1,2-dehydroreticuline synthase, DRR—1, 2-dehydroreticuline reductase, SalSyn—salutaridine synthase, SalR—salutaridine reductase, SalAT—salutaridinol 7-O-acetyltransferase, THS—thebaine synthase, T6ODM—thebaine 6-O-demethylase, COR—codeinone reductase, CODM—codeine O-demethylase, BBE—berberine bridge enzyme, SMOT—(S)-scoreline-9-O-methyltransferase, CAS—canadine synthase, TNMT—tetrahydroprotoberberine N-methyltransferase, 7OMT—7-O-methyltransferase, TPOX—tetrahydropapaverine oxidases, 3OMT—3-O-methyltransferase, OMT—O-methyltransferase.

The biosynthetic pathway is responsible for the synthesis of pharmaceutically significant compounds: papaverine, noscapine, thebaine, morphine and codeine (figure 1).

### 3.1. (S)-reticuline: a central intermediate

Benzylisoquinoline is derived from L-tyrosine, which is synthesized through the shikimate pathway by dehydrogenation and decarboxylation of arogenate [40]. L-tyrosine makes two substrates: 4-hydroxyphenylacetaldehyde (4-HPAA) and dopamine. There are two different pathways to generate dopamine from L-tyrosine i.e. decarboxylation and oxidation. In the presence of enzyme tyrosine decarboxylase, tyrosine is converted into tyramine [41], which is then further oxidized by polyphenol oxidase and outputs dopamine or can be oxidized by tyrosine hydroxylase and form 1-3,4-dihydroxyphenylalanine (L-DOPA) [42] which is then followed to decarboxylation by DOPA decarboxylase to render dopamine. On the other hand, tyrosine undergoes transamination by tyrosine transaminase and is converted into 4-hydroxyphenyl pyruvate (4-HPP) which is then decarboxylated by 4-HPP decarboxylase to provide 4-HPAA [43]. BIA synthesis begins with the condensation of these two derivatives to yield (S)-norcoclaurine coupled with the enzyme norcoclaurine synthase (NCS) [44]. NCS belongs to a pathogenesis-related protein family (PR)10 [1,45].

(S)-norcoclaurine is a central intermediate of the biosynthetic pathway. (S)-norcoclaurine converts into (S)-coclaurine followed by (S)-N-methylcoclaurine through methylation of 6-O-methyltransferase (6OMT) and coclaurine N-methyltransferase (CNMT) [46,47], yielding (S)-N-methylcoclaurine. The hydroxylation (3-hydroxylation) of (S)-N-methylcoclaurine to 3-hydroxy-N-methylcoclaurine is catalysed by a cytochrome P450-dependent monooxygenase [(S)-N-methyl coclaurine 3-hydroxylase (NMCH)] [48,49]. Consequently, 4-O-methyltransferase (4OMT) converts 3-hydroxy-N-methylcoclaurine to (S)-Reticuline [46]. It is the least common intermediate that involves a highly specific enzymatic route and synthesizes more complex structures like protoberberines, benzophenanthridines and morphinans.

#### 3.1.1. Papaverine

Papaverine contains an O-linked methyl group at the C6, C3, C7 and C4 positions. There is limited and controversial information available for the hypothetical pathway for papaverine biosynthesis [50]. Two pathways have been suggested; first is N-methylated (the NCH_3_) pathway including (S)-reticuline from which morphine, codeine, thebaine and noscapine are also synthesized and the second is N-desmethylated (NH) pathway including the (S)-norreticuline. The N-methylated pathway was proposed based on the research using heavy isotope-labelled precursors [36] which starts from the catalysis of (S)-reticuline by enzyme reticuline 7-O-methyltransferase (7OMT) yielding (S)-laudanine [47]. Subsequently (S)-laudanine is transformed to (S)-laudanosine catalysed by 3-O-methylations (3OMT). Afterwards, (S)-laudanosine N-methylated yields tetrahydropapaverine. Likewise, the N-desmethylated (NH) pathway was supported by feeding experiments, gene suppression and radioactive precursor labelling of papaverine synthesis [51]. (S)-coclaurine works as a branch point. 3-hydroxylation forming (S)-6-O methyl norlaudanosoline from (S)-coclaurine followed by O-methylations and yields norreticuline. Subsequently, norreticuline-7-O-methyltransferase (N7OMT) catalyses the norreticuline to norlaudanine followed by3OMT and yields tetrahydropapaverine. Tetrahydropapaverine is common in both the proposed pathways, which is consequently dehydrogenated to dihydropapaverine and papaverine. Recently tetrahydropapaverine oxidase, a flavoprotein oxidase has been confirmed to dehydrogenate (S)-tetrahydropapaverine yielding papaverine [52]. Therefore, papaverine is synthesized by 3-O-methylation, N-demethylation and dehydrogenation.

#### 3.1.2. Noscapine

Noscapine belongs to the structural subgroup of BIAs, phthalideisoquinoline alkaloids. Noscapine biosynthetic route comprises the embodiment of benzylisoquinoline moiety explained by Battersby through the experiment with [^14^C] Tyr and [^14^C] norlaudanosoline [53]. Initial steps involve the berberine bridge enzyme [54,55] in the alteration of the (S)-reticuline to (S)-scoulerine. Afterwards, the enzyme (S)-scoreline-9-O-methyltransferase (SMOT) catalyses (S)-scoulerine to (S)-tetrahydrocolumbamine, which then forms a methylene bridge for the conversion of (S)-canadine by enzyme canadine synthase, a methylenedioxy bridge-forming P450-dependent monooxygenase. (S)-canadine is also known as (S)-tetrahydroberberine which acts as a substrate for tetrahydroprotoberberine oxidase (STOX) enzymes. Subsequently, the N-methylation of (S)-canadine by tetrahydroprotoberberine N-methyltransferase (TNMT) produces (S)-N-methylcanadine. The pathway proceeds with the hydroxylation of (S)-N-methylcanadine at C-1, then methylation by an O-methyltransferase (OMT) to form the compound (S)-1-methoxy-N-methylcanadine which is then oxidized by several steps to yield noscapine.

#### 3.1.3. Morphine, codeine and thebaine

The synthesis of morphine and codeine begins with the isomerization of (S)-reticuline with 1,2-dehydroreticuline synthase enzyme forming the intermediate 1,2-dehydroreticuliniumion, followed by stereospecific reduction through 1, 2-dehydroreticuline reductase which yields (R)-reticuline [56,57]. Then, the C-C phenol coupling of (R)-reticuline is catalysed by the salutaridine synthase (SalSyn) enzyme (the P450-dependent enzyme CYP719B1) yielding salutaridine, which is then converted by salutaridine reductase (SalR) resulting in the formation of 7(S)-salutaridinol [58]. Afterwards, by salutaridinol 7-O-acetyltransferase (SalAT), the acetylation of the hydroxyl group of 7(S)-salutaridinol leads to the formation of salutaridinol 7-O-acetate, which in turn undergoes spontaneous rearrangement by thebaine synthase to give pentacyclic thebaine [59,60]. The pathway separates at thebaine and produces two by products: neopine and oripavine which are synthesized via the multi-step transformation of thebaine. Neopinone catalysed by thebaine 6-O-demethylase (T6ODM) is spontaneously rearranged to codeinone [61,62]. The codeinone reduces to codeine by NADPH-dependent codeinone reductase (COR) enzyme, which is converted into morphine by O-demethylation through codeine O-demethylase (CODM). In the alternative pathway, thebaine catalysed by CODM yields oripavine which is finally reduced by COR to morphine [51].

## 4. Pharmacological action of opioid on neural cell membrane

Opium and its derivatives have traditionally been used as a painkiller to alleviate moderate to severe pain. Opium acts directly on the central nervous system (CNS) [63]. Signal transduction is prevented due to molecular and cellular changes in the pain-signalling neurons when opioids bind to opioid receptors inside the CNS. Opioid drugs imitate endogenous peptides which are naturally produced in the body for small pain-killing effects. Opioid drug binding strength and durability are more pronounced and widespread than endomorphins or natural signalling molecules. Opioid drugs explain their function through an endogenous opioid system which consists of three receptors, situated in the brain named µ (Mu opioid recetor (MOR)), *δ* (delta opioid receptor (DOR)) and *κ* (kappa opioid receptor (KOR)) opioid receptors [64]. These are the component of the superfamily G inhibitory protein-coupled 7-transmembrane receptor (GPCR) [65].

Opioid receptors are distributed throughout CNS in peripheral nerve endings (Mu opioid receptor (MOR)), spinal cord and periaqueductal grey (PAG) (DOR), in the midbrain and brainstem, also in the medulla, hypothalamus and amygdala (KOR) [66]. Studies suggest that MOR is an essential and single-molecular target that mediates the therapeutic effect and the adverse effect of opioids [67]. The role of MOR in morphine's pharmacological effect has been shown in experiments with transgenic and knockout mice [68,69]. Endomorphin (MOR agonist), Enkephalin (DOR) and dynorphin (KOR) are the main endogenous ligands. Both endorphins and opioid drugs can bind to the body's receptors, but opioid drugs bind more strongly and for a longer period than endorphins, which makes them more useful in managing extreme pains like in cancer treatment.

**Figure 2.**
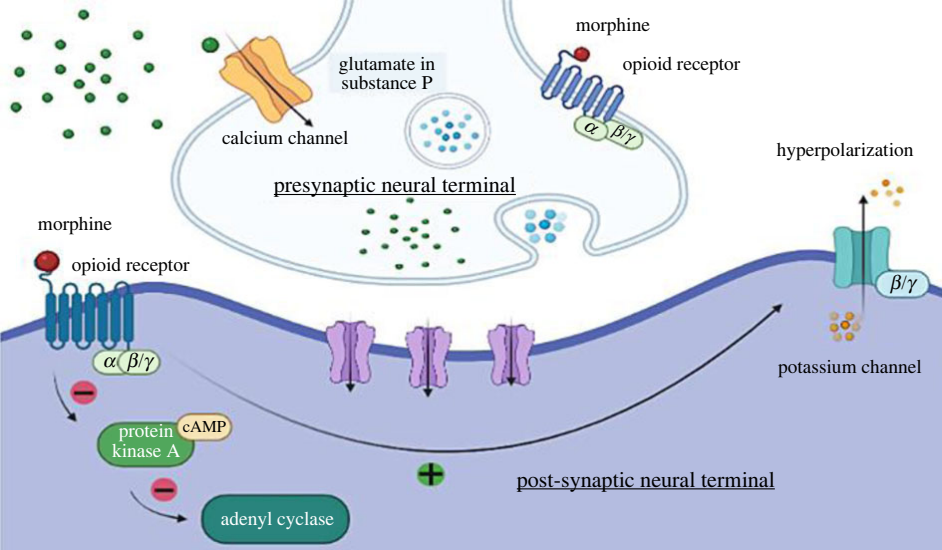
Mechanism of opioid binding to the target Mu opioid receptor at neural terminal.

Activation of G protein including b-arrestin binding results from various changes at the molecular and structural level on the binding of the opioid drug to the receptor. G proteins are composed of free non-identical subunits *α*, *β* and *γ*. In presynaptic inhibition, the activated G-protein in the *α* subunit interacts with other cell proteins after dissociating from the *βγ* heterodimers [70]. On the other hand, *βγ* subunit after release prevents the opening of nearby voltage-gated calcium channels by interacting with them. Neurotransmitters are not released without the calcium influx. In case of postsynaptic inhibition, *βγ* subunit opens the potassium channels by interacting with them. Due to this, the positively charged potassium ions flow out through the channel (figure 2). G proteins have different classes of G*α* subunits whose function is to stop cyclic adenosine monophosphate (cAMP) synthesis and inhibit adenylyl cyclase. To activate or inhibit the signalling pathway cAMP-dependent protein kinase which is activated by cAMP, phosphorylates the multiple neuronal proteins. Opioids relieve pain by switching descending pathway over the ascending pathway [70].

When an opioid binds to an opioid receptor, it stimulates dopamine release, which gives the feeling of pleasure and suppresses the noradrenaline release simultaneously. Eventually, the body becomes tolerant to opioids. For the rewarding effect of the same release of dopamine, people have to take high doses to achieve the same pharmacological effect that leads to physical dependence and addiction. To maintain a new balance, the body increases the number of noradrenaline receptors for functioning normally. Concurrently, due to the suppression of noradrenaline, the body experiences many side effects like constipation [71], wakefulness, indigestion and blood pressure [72]. If an opioid-addicted person stops taking them suddenly, then the person starts showing withdrawal symptoms.

## 5. The potential link between opium and cancer

Opioids are available both legally and illegally. The opioid crisis began in the 1980s and 1990s when pharmaceutical companies started marketing opioids as a painkiller without considering the addictive potential and underestimating their side effects to both the medical community and public. Since then, the crisis of opioid addiction continues till now [73]. For pain management of cancer, opium and its derivatives are extensively used. Throughout history, opium is considered a widely abused and deadliest drug [74]. Currently, the world is experiencing an opioid epidemic era and the number of overdoses is increasing day by day.

Cancer is characterized as a rapid and abnormal cell growth that has the potential to spread to different organs of the body, also known as neoplasia or malignancy. The number of risk factors for developing cancer is attributed to environmental agents, lifestyle-related factors [75,76] and behavioural factors [77]. In behavioural factors, opium addiction and smoking are primary risk factors for several cancers [78]. Previous studies suggest that opium consumption or opium addiction is among the major risk factors that are linked with certain cancers including lung [79], oesophageal [80], pancreatic [81], gastric [82,83], laryngeal [84] and bladder [85]. Rashidian *et al*. [86] studied the potential link between the use of opioids and the incidence of cancer in high-risk areas of the world.

Many case–control, cohort and epidemiological studies have been conducted in recent years to show the role of opium in cancer, which have provided evidences, suggesting that the use of opium alkaloids may raise the risk of various tumours [87] (table 2). Bladder cancer is the most common malignancy of urogenital carcinoma worldwide [95,96]. In recent years, the cases and mortality rates due to bladder cancer have risen in various countries, whereas the study reveals the maximum death rates are detected in Middle Eastern countries and North Africa [97]. Smoking opium is the key risk factor for developing bladder cancer, especially urothelioma or transitional cell carcinoma which is the widespread cancer type. Due to recreational exposure to cigarette smoking, the risk factor of bladder cancer in males is more than in females [98]. In an epidemiological study, Hosseini *et al*. suggested that the bladder cancer possibility increases five times more in the opium consumer population. All digestive tract-related malignant diseases are considered gastrointestinal (GI) cancer which includes esophageal, pancreatic, gastric, hepatic, gallbladder and colorectal cancer [88]. In an epidemiological case–control study, Shakeri *et al*. [99] found that an emerging esophageal cancer cell carcinoma risk factor can be opium. To evaluate the association between opium use and cancer risk, the meta-analysis review of 21 observational studies, with a combined sample size of 64 412 individuals and 6658 cases of cancer was carried out by Mansouri *et al*. [13]. The analysis found that individuals who had ever used opium had a 3.53 times greater risk of developing any type of cancer, compared to those who had never used opium [13]. On the other hand, by meta-analysis investigation, the relationship between opium consumption and bladder cancer was found by studying 11 case–control, five cross-sectional and one cohort case. The study found that the odds of developing bladder cancer were 3.85 times higher for those who used opium alone and 5.7 times higher for those who used both opium and cigarettes. Also, the ratio of bladder cancer development was estimated to be 5.3 times higher for those who used opium [85]. A study reported in IARC monograph 2021, a population-based cohort of 50 045 individuals aged 40–75 years from northeast Iran found that opium use is associated with an increased risk of developing various cancers [100]. However, it should be noted that there is currently no statistical data available from the World Health Organization regarding the link between opium use and cancer.

**Table 2.** Relation of opium to different types of cancers.

addicted drug and adjusted factor	type of study	source of control	key finding	references
**bladder cancer**				
opium consumption, smoking	case–control study	community	smoking and opium are risk factors for bladder cancer	[85]
opium consumption	case–control study	community	opium usage significantly fivefold increases the risk of bladder cancer	[88]
opium consumption	case–control study	community	potential strong risk factor for bladder cancer	[89]
**GI cancer**				
opium use, hookah, cigarette smoking	cohort study	community	high incidence of gastric cancer	[83]
opium and its derivatives	case–control study	community	opium is an important risk factor for colorectal cancer	[90]
opium consumption	cohort study	community	long-term opium use increased the risk of death	[91]
opium use	prospective cohort study	community	opium was notably linked with an increased risk of pancreatic cancer	[92]
opium use	case–control study	clinic	opium consumption and GI cancer formation are positively associated	[93]
**lung cancer**				
opium use and cigarette smoking	case–control study	clinic	smoking opium is associated with a high risk of lung cancer	[79]
opium use	cohort study	community	long-term opium use is associated with increased mortality from both malignant and non-malignant respiratory diseases	[94]

## 6. Possible mechanisms of cancer caused by opium

To show the correlation between opium use and cancer, two mechanisms have been observed in literature which include exposure to opium smoke or pyrolysate and alkaloid constituent of opium [77,86]. Both contain probable carcinogenic and high mutagenic compounds. In the case of opium smoke, the individual heats the drug at a high temperature to vaporize or pyrolyze its active compound and uses a special pipe to inhale smoke. The inhaled smoke contains polycyclic aromatic hydrocarbon compounds which can show carcinogenic effects. Subsequently, after inhalation by individual, the residual component is scraped and eaten without any refinement. This residual component showed a mutagenic effect in various studies [101,102]. Nitrogen-containing heterocyclic constituents are the main carcinogenic compounds obtained from the pyrolysis of morphine. In the second case, the body absorbs constituents of alkaloids derived from opium [103]. Although the evidence of a correlation between both is limited, however, numerous researches indicate a relationship between them [104].

Programmed cell death also known as apoptosis plays an essential role in the growth and control of neoplasm [105]. Apoptosis occurs via caspase cascade activation and is regulated by two distinct pathways, called the extrinsic pathway (death receptor-mediated pathway) and intrinsic pathway (mitochondrial-mediated pathway). The extrinsic pathway is initiated by the ligation with other cells, generally by subsets of T lymphocytes [106] and the intrinsic pathway begins with signals from within the cell. Balance is maintained by anti-apoptotic (Bcl-2 and Bcl-x) and proapoptotic (Bax and Bak) protein sets which are present in the mitochondrial membrane to regulate the intrinsic pathway [107]. The cancer cells can block apoptosis which helps them to survive and replicate. Opioid binds opioid receptors and activates PI3K/Akt pathway which mediates the anti-apoptotic effect [108].

**Figure 3.**
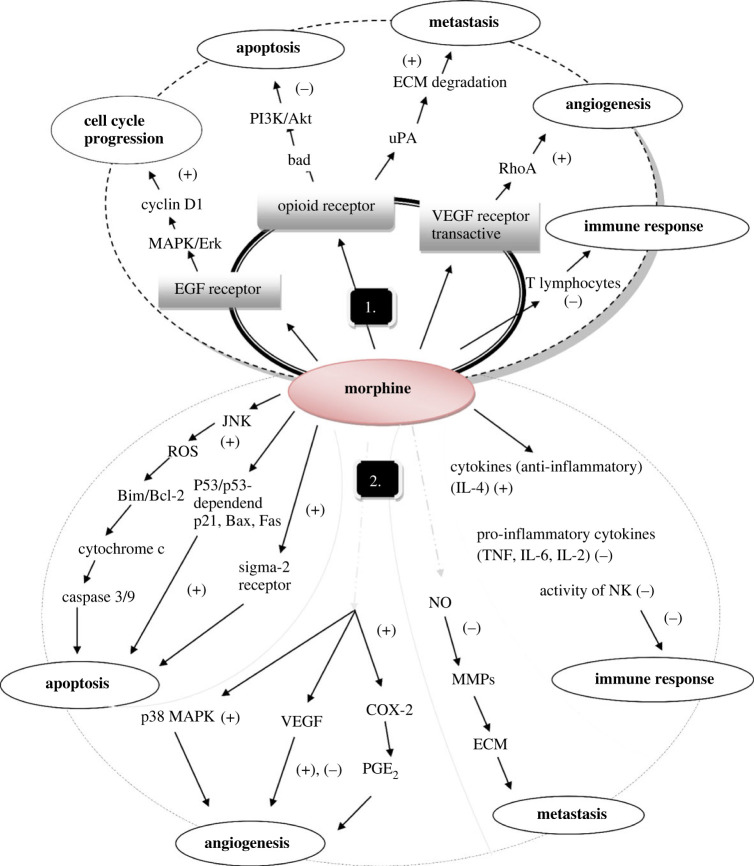
Morphine binds to MOR: (1) possible mechanism and (2) possible pathway affects tumour progression and suppression. (+) Stimulation, (−) Inhibition.

The tumour is initiated when its cells produce several proteins which stimulate the blood vessel development around the tumour and this process is known as angiogenesis. Vascular endothelial growth factor (VEGF) is among the key pathways involved in angiogenesis. Opioid binds to opioid receptor and trans-activate VEGF receptor [109]. It induces and invades specific proteins which rupture the basement membrane to let endothelial cells migrate by activating the extracellular kinases and Mitogen-activated protein kinase (MAPK) signalling pathways. MOR (morphine) cross-activates the epidermal growth factor which stimulates MAPK/Erk pathway [110]. These proteins include matrix metalloproteinases and urokinase-type plasminogen activator (uPA) and its receptor UPAR as well as the tissue type plasminogen activator [111]. Morphine also suppresses T-lymphocytes function which leads to immunosuppression [112] (figure 3).

Gupta *et al*. studied morphine-induced tumour progression in an orthotopic mice model (MCF-7 cells) of breast tumour obtained in clinically relevant doses of morphine. The result indicates that morphine inhibits apoptosis, increases angiogenesis, promotes cell cycle progression and is potentially dangerous for patients suffering from angiogenesis-dependent cancers. Morphine promotes tumour angiogenesis at clinically relevant doses and increases proliferation and migration in breast cancer [113].

## 7. Merits and demerits of opioid alkaloids

Opium and its derivatives have various effects such as analgesia, sedation, euphoria, respiratory depression, cough centre suppression, temperature regulatory centre suppression and vasomotor centre suppression (table 3). Morphine is the principal alkaloid of opium and codeine is methyl morphine. Opium is an excellent painkiller, that is used for symptomatic relief or excruciating pain like myocardial infarction, emergency crush injury and cancer pain [117]. It prevents neurogenic shock, other autonomic effects and is also used as pre-anaesthetic medication and surgical analgesia [118]. Other effects of morphine include constipation by acting on GI smooth muscles, suppression of hypothalamus leading to decreased anti-pituitary hormones, hypotension and constriction of pupils miosis. In addition, adverse effects of morphine include nausea, vomiting, abdominal pain, constipation and other symptoms such as urinary retention and urgency, respiratory depression and in some cases allergy. The toxic effects of morphine starts at doses above 50 mg and the lethal dose of morphine is 250 mg. Morphine causes the release of histamine which causes worsening of bronchoconstriction. Due to prolonged use, morphine resistance is developed by the target receptor so the euphoric effects and a few other effects will not take place. Codeine is less potent than morphine. Sixty milligrams of codeine produces the same analgesic effect as 600 mg of aspirin. There are certain enzymes known as CYP2D6 that act on codeine which causes demethylation and converts codeine into morphine giving the same effect on the body as normal morphine. Codeine has a more selective cough suppressant action so it is used in the treatment of cough but it also causes constipation as a side effect. This side effect can be used to treat diarrhoea and euphoria. Papaverine is commonly used as a vasodilator or a smooth muscle relaxant during microsurgery and as an antispasmodic drug to cure migraine headache, schizophrenia, renal, biliary colic, intestinal and urinary tract spasms. The side effects of papaverine include drowsiness, skin rash, abdominal distress and anorexia.

**Table 3.** Pharmacology of alkaloids from *Papaver somniferum.*

alkaloids	chemical formula	structural formula	effect on the human body	pharmaceutical use	references
morphine	C_17_H_19_NO_3_	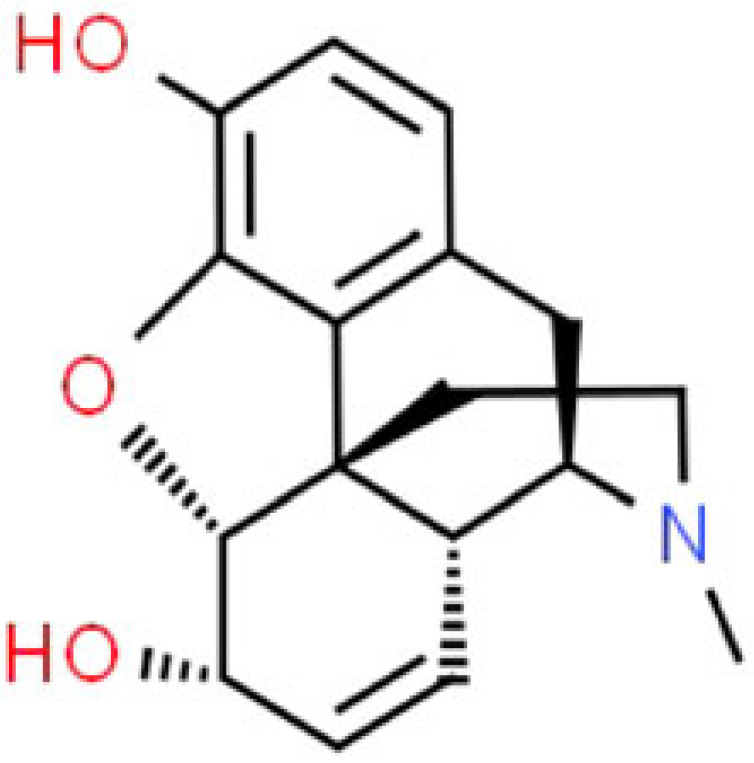	morphine consumption can lead to severe hypotension by decreasing systemic arterial pressure temporarily caused by a reduction of vascular resistance	analgesia, general anaesthetic, cough suppressant, anti-diarrheal, relieving pain of myocardial infarction	[71]
codeine	C_18_H_21_NO_3_	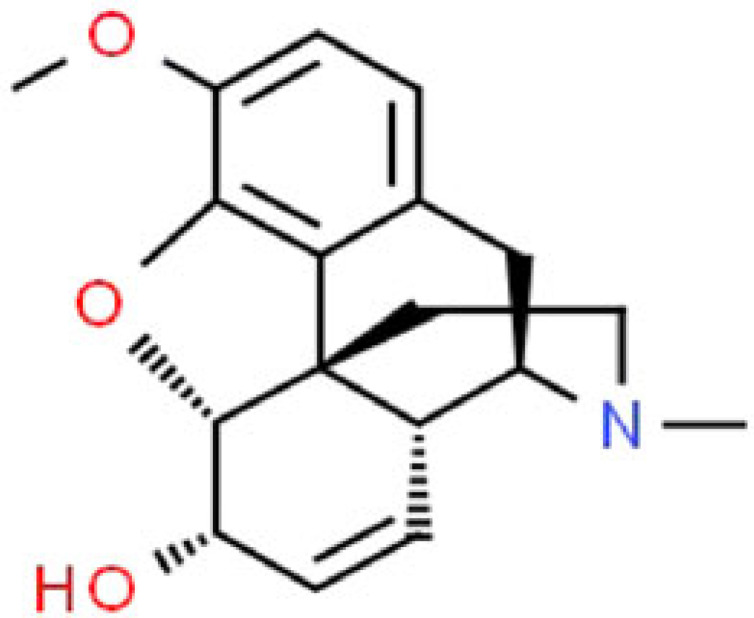	changes how our body feels and our brain responds to pain	helps to relieve mild to moderate pain	[72]
thebaine	C_19_H_21_NO_3_	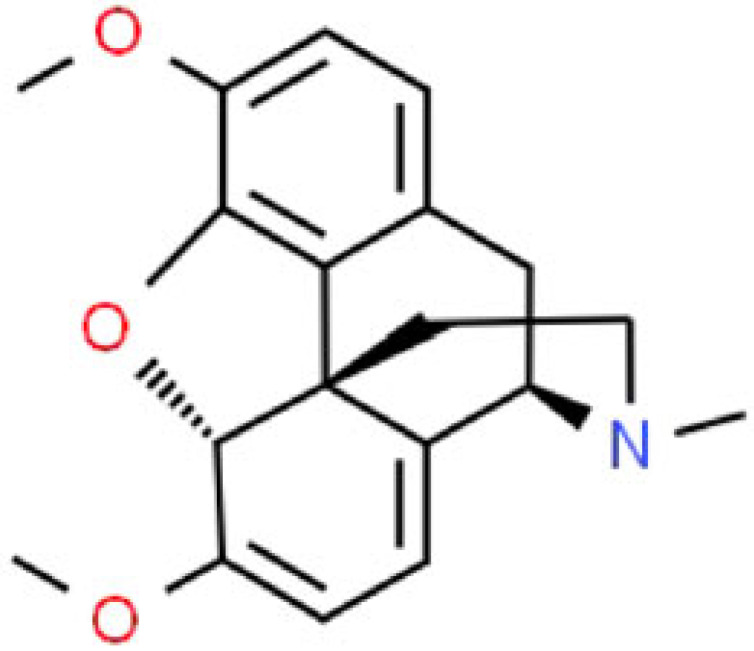	the raw material for the synthesis of oxycodone, oxymorphone, buprenorphine, naloxone and related semi-synthetic opiates	used in the pharmaceutical industry for the synthesis of oxycodone, oxymorphone, buprenorphine, and naloxone	[114]
narcotine (noscapine)	C_22_H_24_NO_7_	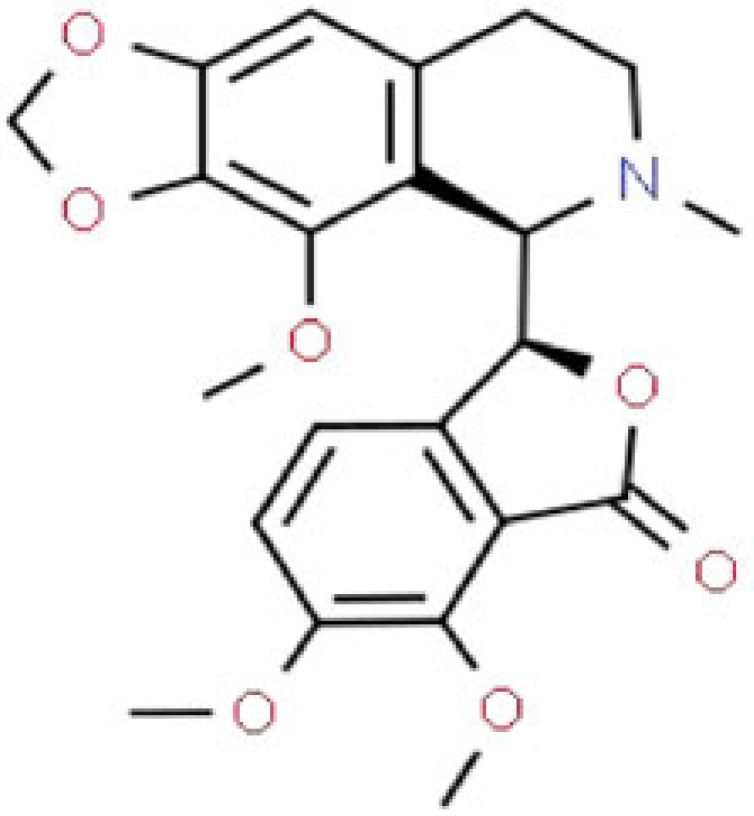	applies its antitussive effects and also exerts an antimitotic effect	mild analgesic, antitussive (cough-suppressing) effects, potential antineoplastic activities (anticancer activity)	[115]
papaverine	C_20_H_21_NO_4_	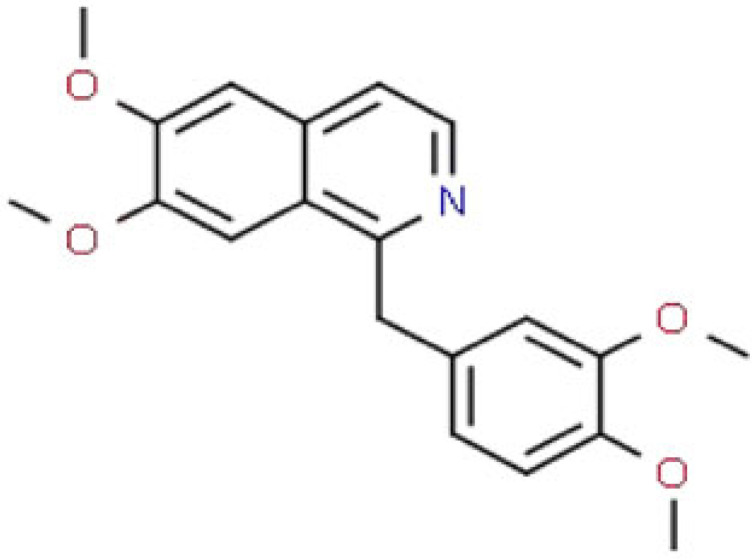	relaxes various smooth muscles	vasodilator	[116]

## 8. Conclusion

*Papaver somniferum* is a natural source of BIAs. In addition to being the most disputed drug, opium also has wide pharmaceutical importance. For the therapeutic purpose, it is necessary to understand the extraction and analysis process, its biosynthetic pathway and its effect on the human body. Details on biosynthetic pathways of opium alkaloids and related enzymes have not yet been elucidated and more comprehensive work is required which will help in determining the impact of opium on the human body. Furthermore, based on previous studies carried out, regarding the analysis and quantification of individual alkaloids through different techniques, HPLC is more frequently used technique due to its efficiency and accuracy.

In case of opium and cancer correlation, recent studies have shown that morphine has a role in tumour progression. By smoking or ingesting, opium alkaloid users get exposed to several toxicants and carcinogens. Due to the calming effect, opioid painkillers have a very high rate of abuse which can lead to addiction. Opium addiction, misuse and overdose are independent risk factors for various cancers. However, the mechanism of action of opiate addiction remains unclear.

## 9. Future aspects

Opium is a highly effective pain reliever used to treat severe pain from conditions including myocardial infarction, acute crush injuries and cancer pain. More future studies are required in the field of pathway analysis, quantification methods and the mechanism of cancer by this family of alkaloids. Considering the hypothetical papaverine biosynthetic pathways, there is insufficient and controversial information available. Also, further research is required to recognize the molecular mechanism and effect of morphine on tumour progression. Recent case–control, cohort and epidemiological investigations on the relationship between opium use and cancer have revealed evidence that using opium alkaloids may increase the chance of developing a number of tumours. It is still unclear how tumour cell proliferation is controlled and how morphine affects it.

## Data Availability

This article has no additional data.
